# Emerging challenges and opportunities in innovating food science technology and engineering education

**DOI:** 10.1038/s41538-023-00243-w

**Published:** 2024-01-13

**Authors:** I. S. Saguy, C. L. M. Silva, E. Cohen

**Affiliations:** 1 The Robert H. Smith Faculty of Agriculture, Food & Environment, The Hebrew University of Jerusalem, Rehovot, Israel; 2 Universidade Católica Portuguesa, CBQF—Centro de Biotecnologia e Química Fina—Laboratório Associado, Escola Superior de Biotecnologia, Rua Diogo Botelho 1327, 4169-005 Porto, Portugal; 3 Gilford Glazer Faculty of Business Administration, Ben-Gurion University of the Negev Beer-Sheva, Be’er Sheva, Israel

**Keywords:** Education, Agriculture

## Abstract

Progress in science, technology, innovation, and digital capabilities call for reassessing food science, technology, and engineering (FST&E) education and research programs. This survey targeted global professionals and students across food disciplines and nutrition. Its main objectives included assessing the status of FST&E higher education, identifying challenges and opportunities, and furnishing recommendations. Seven topics affecting the future of the FST&E curricula were evaluated by the panel as ‘High’ to ‘Very high’, namely: ‘Critical thinking’, followed by ‘Problem-solving projects’, ‘Teamwork/collaboration’, ‘Innovation/Open innovation’ and ‘Multidisciplinary’. The importance of academic partnership/collaboration with the Food Industry and Nutrition Sciences was demonstrated. Significant positive roles of the food industry in collaboration and partnerships were found. Other essential food industry attributes were related to internships, education, strategy, and vision. Collaboration between FST&E and nutrition sciences indicated the high standing of this direction. The need to integrate or converge nutrition sciences and FST&E is emphasized, especially with the growing consumer awareness of health and wellness. The study provides insights into new education and learning opportunities and new topics for future curricula.

## Introduction

The unabated progress in science, technology, and innovation, combined with the exponential rate of change facilitated by the proliferation of computerized capabilities and artificial intelligence (AI), calls for reassessing the food science, technology, and engineering (FST&E) education. The fourth industrial revolution (i.e., Industry 4.0) highlights significant progress in numerous fields, including robotics, smart sensors, AI, the Internet of Things (IoT), big data, cloud computing, safety, and production efficiency^1^. Climate change, global population growth, high levels of food loss and food waste, and the risk of new disease or pandemic outbreaks are examples of numerous challenges that are potential threats to future food sustainability and the security of the planet that urgently need to be addressed^2^.

The projected global population growth reaching 10 billion people by 2050 highlights the acute need for new evaluations of FST&E education system background to address mounting challenges and opportunities. The complexity and predicted immense size of future tasks call for new paradigms, an open innovation mentality, and a novel mindset promoting multidisciplinary collaborations and partnerships^3^.

Disruptions such as digital agriculture, the fourth industrial revolution (industry 4.0), food agility, big data, and AI have been utilized to characterize the changes in the way agro-food systems evolve and function, as well as in the approach they have been analyzed, measured, and monitored^4^. For instance, Wageningen University, one of the leading influential universities, has also taken an active strategy to align with the developments in IT and AI. Apart from the content-wise shift, skills such as critical thinking, creativity, and problem-solving are addressed by applying project-based evaluations^5^. The industrial revolution (industry 4.0) and moving to industry 5.0 include new enabling technologies (e.g., big data, IoT, cloud computing) besides AI, digital twins, machine learning, virtualization, and others^6^.

Food science and technology (FST) and especially food engineering (FE) in academia face diminishing funding for research, dwindling critical masses in faculties (particularly at universities in the USA), decreasing student enrollment^7^ and impacting future cooperative extension education and research needs^8^. This leads to the observation by some food-related education programs to be at a crossroads and the need to reassess their vision and expand the scope to grand societal drivers such as health and wellness (H&W), the mutual host and the microbiome considerations, food security and safety, population growth, aging, water and land scarcity, and environmental concerns^9^. Other reasons for integrating stakeholders outside the food manufacturing industry have been proposed^10,11^. Members of the FST&E professions request a broader and more applied education that offers better opportunities for entrepreneurship^12^ .

FST&E professions are witnessing significant challenges as well as changes imposed by the accelerated rate of change and digital transformation. The expected changes will most probably affect FST&E education as already projected previously^7,10–15^. This forward-looking, combined with the radical changes witnessed during and post-COVID-19, calls for a change in traditional education and curricula paradigms. For instance, the new vision deploys concepts of FST&E in the context of sustainable food processes, products for changing lifestyles and beliefs, innovation for H&W, and novel methodologies that suit audiences of the digital age. Courses on entrepreneurship and innovation, novel education methods, and enforcing quality standards and certification have been also proposed for Europe^14^.

Engineering education is also experiencing dramatic changes. The traditional teaching model, where students are passive in the lecture room, gives way to more active, student-centered, and participatory approaches. Different modern education and learning techniques, such as blended and flip-classroom, active learning, use of technology in teaching, universal design, and student-centered education approach, among others, were previously reported^10^. For instance, active learning utilizing a teaching app called TopHat (https://tophat.com/) to administer a daily quiz, encouraged group work and discussion, and peer evaluation was also reported^16^.

Active engineering learning promotes the acquisition of knowledge and essential soft skills such as teamwork, problem-solving abilities, and entrepreneurial mindsets^17^. It also encourages the utilization of digital technologies such as simulation software and virtual laboratories^17^. It is worth noting the pioneering virtual experiments and laboratories in food science, technology processing, and engineering area^18^.

Among novel methodologies suggested for engineering education are project-based learning, hybrid learning, the flipped classroom, and design thinking^10,19–21^ .

The role of the food industry and other related sectors in contributing to and assisting educational institutions in designing curricula that provide the skills demanded by the job market was highlighted recently. It emphasized that current Bachelor´s and Master´s degrees follow programs that attempt to offer a practical perspective but still focus on the academic point of view. To bridge the gap between academia and industry, the University Extension Diploma in Food Technology (DEUTA) deepens into the food sector, seeking professional qualifications for participants. This is achieved by both first-hand know-how of food sector professionals and academics, along with an internship period in a food company. Collaborative courses strengthen academia-industry bonds, and employability is boosted thanks to internships and the network created^22^.

Innovation and entrepreneurship are key factors to provide added value for food systems. Based on the findings of the Erasmus+ Strategic Partnership BoostEdu (https://erasmus-plus.ec.europa.eu/ assessed May 16, 2023), three knowledge gaps were reported: (1) identify the needs for innovation and entrepreneurship (I&E) in the food sector; (2) understanding the best way to organize learning; (3) providing flexibility in turbulent times. The results of the project, in particular during the COVID-19 pandemic, highlighted the need for flexible access to modules that are complementary to other sources and based on a mix of theoretical concepts and practical experiences. The main lessons learned concern the need for co-creation and co-learning processes to identify suitable practices for the use of innovative digital technologies^23^. However, there are experts objecting to entrepreneurship courses being a subject of FST&E curricula or that the curricula should be supported with outside presentations or invited talks on this topic. This contrary position could be probably explained by the contrast between academia and more applied and industrial occupations. As the vast majority of the FST&E graduates are employed in various businesses where innovation and startup activities are becoming essential, entrepreneurship aspects should be considered in future education.

New platforms, such as massive open online courses (MOOCs), webinars, blogs, Facebook, Instagram, and Twitter, have opened up new spaces for disseminating ideas, experiences, and training in food-related matters^24^. Online and open learning permits access anytime and anywhere to formal classes, education modules on specific topics, and informal discussion sites^24^. Thus effectively democratizing learning, disseminating knowledge to vast audiences, and coping with the educational demands during the COVID-19 pandemic^25^.

The overall objectives of this study were: 1. Assessing the current status of FST&E education by using a computerized global survey; 2. Identifying current challenges and opportunities; and 3. Suggest recommendations (if needed) for additional directions and topics for future curricula.

## Results and discussion

### Respondents

The total number of respondents that started the questionnaire was 1022. Of these, 703 (68.8%) respondents (the panel) completed the survey. Data from respondents who failed to address all questions and had several missing values were omitted, as they ignored or preferred not to answer some of the questions. The relatively high number of excluded respondents was probably due to the language barrier. Although not explicitly asked, based on respondents’ IP addresses, 88 countries participated in the survey. The overall time for completing the survey was approximately 10–12 min.

### Demographics and geographic distribution

Demographic data are presented in Table 1. The panel was evenly distributed: gender (female/male 1.15:1.00), age (excluding the 18–25 years group, 7.5%). Age distribution indicates good participation of the various groups and experiences.

**Table 1:** Respondents’ demographic.

	Frequency	%
Respondents (panel)	703	100.0
*Gender*		
Male	322	45.8
Female	372	52.9
Others	9	1.3
*Age groups (years)*		
18–25	53	7.5
26–40	180	25.6
41–55	266	37.8
Above 55	202	28.7
*Geographic distribution where the most advanced degree was received or study*		
Western Europe	195	27.7
Eastern Europe	97	13.8
UK	21	3.0
North America, including Canada	92	13.1
Mexico	11	1.6
South America	67	9.5
Asia/Middle East	85	12.1
China	12	1.7
Far East (excluding China)	7	1.0
Oceania (Australia, New Zealand)	12	1.7
Africa	104	14.8

The geographical location of the respondents indicates a global representation, although some regions were more prevalent by the panel. Respondents from China, the Far East (excluding China), and Oceania also participated, but their overall percentage was relatively low (combined value of 4.4%). However, combining Asia and the Middle East respondents resulted in a significant representation (16.5%). The surprising outcome was the high number of African respondents (14.8), probably due to the good network of IUFoST contacts in Africa. Although participation was quite impressive in terms of global feedback (88 countries), the number of respondents in a specific region was quite low in some cases, and consolidation was necessary for further analysis. Nevertheless, the widespread number of respondents from a wide spectrum of countries demonstrated that the survey had a global distribution, offering a significant improvement compared with a previous study^15^.

### Main professional activities and education

The panel (703 respondents) professions consisted of food scientists and technologists (FSTs) 398 (56.6%), food engineers (FEs) 120 (17.1%), microbiologists (HMs) 25 (3.6%), nutritionists (HNs) 35 (5.0%), chemical engineers (CEs) 19 (2.7%), bioengineering/biotechnology (BBs) 7 (1.0%), business/marketing (BMs) 14 (2.0%), consultants (COs) 41 (5.8%), and others (food trade company, regulators, etc.) 41 (5.8%). As 73.7% of the respondents were FSTs and FEs, students, and graduates, the data reflect professional positions within FST&E disciplines, as was also previously shown^15^.

**Fig. 1:**
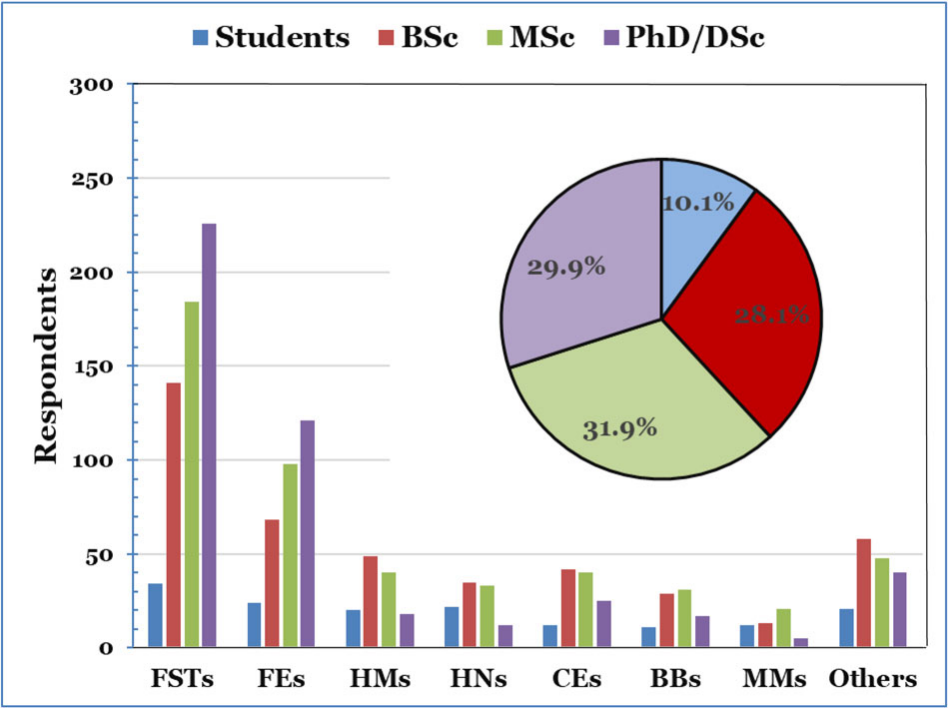
Respondents education fields: food scientists and technologists (FSTs), food engineers (FEs), microbiologists (HMs), nutritionists (HNs), chemical engineers (CEs), bioengineering/biotechnology (BBs), business/marketing (BMs), and others. Overall degrees distribution (small insert).

The respondents were also asked to fill in all their degrees in the various education categories using up to 4 options (student, BSc/1st Degree, MSc/equivalent, and Ph.D./DSc). Fig. 1 highlights the panel degrees distribution. The relatively high number of doctoral (Ph.D./DSc, 464, 29.9%) is not surprising considering the academic affiliation of most of the respondents (see Section “Affiliation”). It should be noted that many of the respondents hold more than one degree, explaining the high number of overall degrees of the panel (1550), as also depicted in Fig. 1.

### Affiliation

The combined high majority of the respondents affiliated with educational and private research institutes (71.7%) provides a possible explanation for the extra number of doctoral degrees in the panel. Conversely, based on the respondents in the age group 41–55 and above 55 (37.8 and 28.7%, respectively) and the fact that a high percentage of the majority of the respondents hold a doctoral degree, the data are likely to reflect professional middle to high management levels and leadership positions within educational, institutions and possibly in the food industry. It should be noted that the number of respondents from industrial affiliation (food industry, food service, startups/FoodTech, and consultants, excluding government) was quite high (18.2%), probably projecting that although academia and industry are not equally represented, industrial affiliations are well represented (i.e., 128 responders).

### Topics affecting the future of the professional domain curricula

The importance of 10 topics to be included in developing future curricula using the Likert-type scale^26^ was evaluated. The topics listed included post-COVID-2019 considerations and several other new concepts. Table 2 shows that 7 topics were evaluated above 4.0 (‘High’) based on the calculated Likert-type scores average. The highest average scores were: ‘Critical thinking’ (4.50), followed by ‘Problem-solving projects’ (4.44), ‘Teamwork/collaboration’ (4.31), ´Innovation/Open innovation’ (4.29), and ‘Multidisciplinary’ (4.24). These data highlight possible changes that the FST&E domains anticipate in the post-COVID-19 and remote or hybrid education/learning, as well as the further proliferation of innovation and OI.

**Table 2:** Topics affecting the future of the profession curricula in descending order based on Likert-type average (*n* = 703).

Order	Topic	Total panel	FSTs	FEs
1	Critical thinking development	4.50^a^	4.49	4.52
2	Problem-solving projects	4.44^a^	4.45	4.45
3	Teamwork/collaboration	4.31^b^	4.33	4.23
4	Innovation/open innovation	4.29^bc^	4.33	4.20
5	Multidisciplinary	4.24^bc^	4.18	4.18
6	Creativity	4.22^c^	4.22	4.15
7	Project/time management	4.05^d^	4.12^*^	3.93^*^
8	Soft (life) skills	3.90^e^	3.94^*^	3.70^*^
9	Entrepreneurship	3.77^f^	3.79	3.69
10	Business creation/network	3.70^f^	3.72	3.56

Different small letters in the same column represent significant differences between groups (one-way ANOVA with post-hoc LSD test, *p* < 0.05).

*Significant difference (two-side *t*-test between averages in the same row, *p* < 0.05).

It is important to note that business-related topics were evaluated as less important, with Likert-type scores averaging below 4.0. These included: ‘Soft skills’ (3.90), followed by ‘Entrepreneurship’ (3.77), and ‘Business creation/networking’ (3.70). ‘Entrepreneurship’ and ‘Business creation/network’ could bring many benefits, such as fostering innovation, productivity, competitiveness, new business, OI, and socioeconomic development. Yet, these topics were considered among those of less importance, probably indicating that the panel was less oriented to business-related topics.

The search for professionals with different skills to overcome the current and foreseen challenges relevant to the agri-food sector was previously studied^25^. It was shown that problem-based learning (PBL), described as an instructional approach, promotes interdisciplinary and critical thinking with the potential to meet current challenges. PBL, aligned with an innovation program and contest, integrated into a master’s degree in FE to promote academic entrepreneurship, allowed the development of innovative products intending to solve problems faced by the agri-food sector^27^. The latter information supports the current survey data that show that the highest perceived topics were ‘Critical thinking’ (4.50) and ‘Problem-solving projects’ (4.44). On the other hand, the relatively low perceived importance of entrepreneurship (3.77 ranked #9) could indicate that FSs, FTs, or FEs are currently considering business-related topics as a lower priority. Nevertheless, their Likert average scores were approaching ‘High’. It is important to note that promoting project-based learning by students on developing eco-designed business models and eco-innovated food products seems to be an essential lever for the sustainability transition^10^. Although this is just one example, it highlights the importance of project-based learning^27–29^.

Project-based learning is an integrated part of the flipped classroom (FC) model, based on active learning, and consequently attracts much interest. The FC is a form of blended learning (BL) that reorganizes the workload in and outside the classroom and requires the active participation of students in learning activities before and during face-to-face lessons with teachers^10,30^. The FC model has been applied since the 1990s to encourage student preparation before classes: team-based learning, peer or mentor instruction, and just-in-time education, where the teaching information is communicated via electronic means. This allows more class time to be devoted to active learning and formative assessment^31^. A recent study highlighted a case study where an elective FC course on engineering, science, and gastronomy was implemented for undergraduate students that included in-class demonstrations by chefs. New education methodologies call for expanded computational abilities, ample access to online content, active learning, and student-centered approaches^10^.

A comparison between traditional project-based learning and hybrid project-based learning indicated a significant increase in fundamental formative knowledge, enhanced problem-solving abilities, and production of better-performing artifacts regarding the set of design skills for students undergoing hybrid project-based learning^28^.

In light of the feedback by the panel indicating that ‘Critical thinking development’ and ‘Problem-solving projects’ were the highest outcome (#1 and #2, respectively), combined with recent reports on the FC importance, it could be concluded that seeking new directions in learning/facilitating strategies that complement existing methods in order to enrich the learning experience of students is recommended.

### Academic partnership/collaboration

**Fig. 2:**
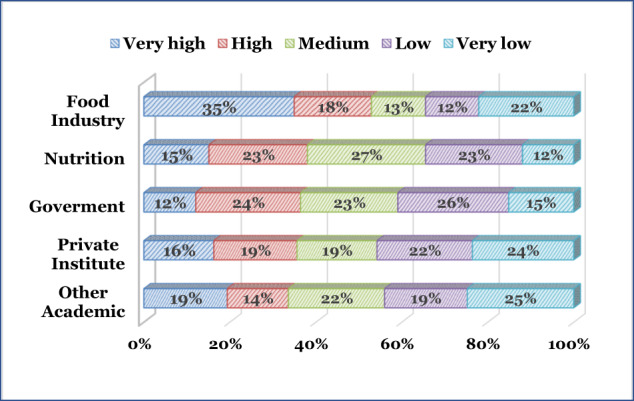
Ranking importance (‘Very high’, ‘High’, ‘Medium’, ‘Low’, ‘Very low’) distribution of ‘Academic partnerships/collaborations’.

The respondents were instructed to rank (from 1 to 5, corresponding to high to low; each rank could appear only once) the importance of partnership(s) and/or collaboration(s) with: ‘Food Industry´, ‘Nutrition sciences’, ‘Government, policymakers and/or local authorities’, ‘Private sector’, and ‘Other academic disciplines’. The ranking distribution is depicted in Fig. 2.

Collaboration with the ‘Food industry’ was ranked the highest, while the collaboration with ‘Other academic programs’ was ranked lower. Furthermore, the top two rankings (‘Very high’ and ‘High’) were ‘Food industry’ (53%), ‘Nutrition’ (38%), ‘Government’ (36%), ‘Private institutes (35%) and ‘Other academic programs’ (33%).

Collaboration with the nutrition sector was highly ranked. This demonstrates that the panel considered collaboration between FST&E and nutrition highly important and is a direction that these domains should consider closely. The need to enhance and probably integrate or converge nutrition sciences and FST&E is underscored due to the lack of present collaboration and the growing consumers’ awareness of H&W and food processing.

The role of the food industry as a key player in academic partnership and collaboration should be considered, particularly due to the negative aspects suggested by the NOVA ultra-food processes food classification. For instance, “*By design, these products are highly palatable, cheap, ubiquitous, and contain preservatives that offer a long shelf life. These features, combined with aggressive industry marketing strategies, contribute to excessive consumption and make these products highly profitable for the food, beverage, and restaurant industry sectors that are dominant actors in the global food system*”^32^. This study demonstrates that the food industry plays significant positive roles in both collaboration and partnerships. It also plays a key part in internships described below (Section “Internships”).

### Topics importance to FST&E

The importance of 11 topics for FST&E was assessed as listed in Table 3.

**Table 3:** Topics importance to FST&E curricula ordered based on the averages obtained by a Likert-type 1–5 scale.

Order	Topic	Total Panel	SD	FSTs	FEs
1	Sustainability, circular economy food waste management	4.44^a^	0.71	4.46*	4.24*
2	Innovation/open innovation	4.36^ab^	0.71	4.39	4.34
3	New product development	4.32^b^	0.78	4.41*	4.20*
4	Consumer perception & trust	4.27^b^	0.79	4.29*	4.08*
5	Nutrition Sciences	4.07^c^	0.82	4.16*	3.82*
6	Startups/FoodTech	3.91^d^	0.82	3.90	3.87
7	Entrepreneurship	3.82^d^	0.90	3.84	3.68
8	Kaizen methodologies/continuous improvement	3.66^e^	0.90	3.65	3.48
9	Artificial Intelligence, machine learning	3.65^e^	0.89	3.60*	3.75*
10	Big Data, communication, robotics	3.58^e^	0.90	3.50	3.57
11	Management/marketing	3.58^e^	0.89	3.59	3.42

Different small letters in the same column represent significant differences between groups (one-way ANOVA with post-hoc LSD test, *p* < 0.05).

*Significant difference (two-side *t*-test between averages in the same row, *p* < 0.05).

The data exposed 5 top important topics to FST&E. The topic of highest interest was ‘Sustainability, circular economy, and food waste management,’ followed by ‘Innovation/open innovation’ and ‘New product development’ (no statistically significant difference between these topics), ‘Consumer perception & trust’ and ‘Nutrition sciences’ that were statistically different from the first two topics (one-way ANOVA with post-hoc LSD test, *p* <0.05), respectively. Worth noting the significant differences between FSTs and FEs in ‘Sustainability, circular economy, and food waste management’, ‘New product development’, ‘Consumer perception & trust’, and ‘Nutrition Sciences’, where FSTs significantly assigned higher importance to these topics in comparison with FEs. However, no significant difference was found for ‘Innovation/open innovation’.

‘Artificial Intelligence, machine learning’ was only ordered as #9 based on the Likert-type scores averages, and FEs considered it significantly higher than FSTs. It is safe to predict that the importance of AI will increase in the coming years once more and more applications and utilizations will emerge. Suffice to consider recent applications and the global AI market size growth from $65.48 billion in 2020, projected to reach $1581.70 billion by 2030, growing at a CAGR of 38.0% from 2021 to 2030 (https://www.alliedmarketresearch.com/artificial-intelligence-market).

### Importance to FST&E curricula to meet future challenges and learning opportunities

The importance of the curricula in meeting FST&E future challenges and learning opportunities (in descending order) is highlighted in Table 4.

**Table 4:** Topics importance in meeting FST&E future challenges and learning opportunities (in descending order based on Likert-type 1–5 scale average).

Order	Topic	Total panel	SD	FSTs	FEs
1	Research project(s)	4.34^a^	0.75	4.42*	4.21*
2	Apprenticeships (e.g., industrial training)	4.28^ab^	0.77	4.29	4.21
3	Adaptability (e.g., adjusting to change in real-time, managing biases, overcoming challenges)	4.22^bc^	0.79	4.21	4.18
4	Revision current programs	4.16^c^	0.79	4.17	4.18
5	Employability	4.13^c^	0.86	4.16	4.02
6	Enhanced integration with nutrition	3.92^d^	0.84	4.00*	4.21*
7-8	Business-related activities (e.g., creation, network, partnerships, collaboration)	3.92^d^	0.91	3.92	3.83
7-8	Soft (life) skills	3.89^d^	0.87	3.95*	3.73*
9	Hybrid teaching	3.78^e^	0.91	3.82	4.21

Different small letters in the same column represent significant differences between groups (one-way ANOVA with post-hoc LSD test, *p* < 0.05).

*Significant difference (two-side *t*-test between averages in the same row, *p* < 0.05).

Table 4 shows five topics were considered to be of ‘Very high’ to ‘High’ importance: ‘Research project(s)’ (4.34), ‘Apprenticeships (e.g., industrial training)’ (4.28), ‘Adaptability (e.g., adjusting to change in real-time, managing biases, overcome challenges)’ (4.22), ‘Revision current programs’ (4.16), and ‘Employability’ (4.13). The other topics received lower scores.

The significant difference between FSTs and FEs on ‘Research project(s)’, ‘Enhanced integration with nutrition’, and ‘Soft (life) skills’ is worth noting. On these topics, except for ‘Enhanced integration with nutrition’, FSTs scores were significantly higher when compared with FEs. The ´Enhanced integration with nutrition´ by both FSTs and FEs was ‘High’ (4.00) and above, projecting the absolute need for FST&E to enhance its collaboration with nutrition, mainly due to the high importance of H&W and its significant role.

Adaptability is the potential to adjust and learn new skills in response to changing factors, conditions, cultures, and environments. It is a soft skill that both colleagues and superiors highly value. In the ever-changing needs and progress, businesses and employees must adapt quickly to unforeseen dynamic circumstances, innovation, and disruption. Adaptability means being flexible, innovative, open, and resilient, particularly under unforeseen conditions. Some key elements of being adaptable are confident but open to criticism, focusing on solutions rather than problems, collaborating with others, and learning from them (https://www.walkme.com/glossary/adaptability/). For instance, the a*daptability* of FST developments implies a capacity to continuously change and improve its operations and food quality output in time and space^33^. This explains the #3 place the panel considered adaptability.

The panel perceived both ‘Revision of current programs’ and ‘Employability’ as high priority (#4 and #5, average of 4.16 and 4.13, respectively). These assessments should be considered carefully by academic programs in order to adapt to the fast changes driven by innovation, disruption, and digital progress.

‘Enhanced integration with nutrition’ came in #6. However, FSTs and FEs indicated this topic is highly important (average of 4.00 and 4.21, respectively). Hence, FST&E education programs should seek avenues to enhance integration with nutrition science. Possible collaborations should consider joint research programs and other partnerships and alliances.

‘Business-related activities (e.g., creation, network, partnerships, collaboration)’ and ‘Soft (life) skills’ were #7–8. Nevertheless, their Likert-type average values were close to ‘High’. Hybrid teaching was perceived as the last (3.78). Apparently, this type of education is not very appealing. Yet, this should be reassessed after the Covid-19 pandemic has passed.

Engineering education is also experiencing dramatic changes. The traditional teaching model, where students are passive in the lecture room, gives way to more active, student-centered, and participatory approaches. Different modern education and learning techniques, such as blended and flip-classroom, active learning, use of technology in teaching, universal design, and student-centered education approach, among others, were previously reported^9^. Hence, it is expected that Hybrid teaching and other advanced methods, including AI, will flourish soon and will become the norm.

### Internships

**Fig. 3:**
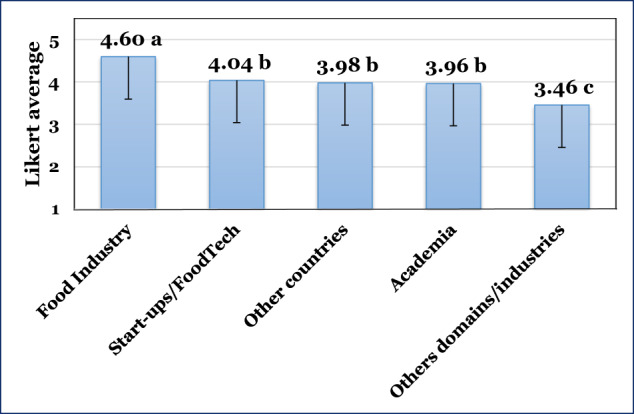
Likert-type averages (1–5 scale) and one side (-) SD of internships importance for FST&E (values with different small letters indicate significant differences between groups; one-way ANOVA with post-hoc LSD test, *p* < 0.05).

The importance of internship to FST&E students was evaluated considering 5 possibilities: ‘Academic internship,’ ‘Food industry internship,’ ‘Start-up/FoodTech company internship,’ ‘Other domains/industries,’ and ‘Internship in other countries.’ The data are depicted in Fig. 3.

The internship was categorized into three statistically different groups (one-way ANOVA with post-hoc LSD test, *p* < 0.05). The first group was internships in ‘Food Industry’ (4.60), followed by the second group: ‘Start-ups/Food Tech’ (4.04), ‘Other countries’ (3.98), and ‘Academia’ (3.96), and the third group ‘Others domains/industries’ (3.46). Comparing the difference between FSTs and FEs, respondents showed a significant difference (one-way ANOVA with post-hoc LSD test, *p* < 0.05) for internships in ‘Food Industry’ (4.65 and 4.52), ‘Start-ups/Food Tech’ (4.11 and 3.89) and ‘Other domains/industries’ (3.46 and 3.26), respectively. It is not surprising that FSTs have consistently assigned higher values to internships, probably due to the possibility that they are more complimentary to hands-on experiences.

Bridging the academia-industry gap in the food sector through collaborative courses and internships was recently studied. More than fifteen years of university extension diplomas in food technology Diplomas demonstrated how collaborative courses strengthen academia-industry bonds, and employability was boosted thanks to internships and the network created^22^. Internships could support students in developing their identity, which is achieved by close contact with their future working tasks^34^, enhancing familiarity with and nearness to their future profession^35^ and industry-based projects and governance^36^. Also, student projects in collaboration with the industry make the students face a reality^37^. In light of these benefits, it is clear why the internship in the food industry received such a high Likert-type average. This very high importance given by the panel to industry internships coincides with their ranking, as aforementioned in the previous section, highlighting the core role of the food industry in students’ education.

### Professional organization impact on FST&E education

**Fig. 4:**
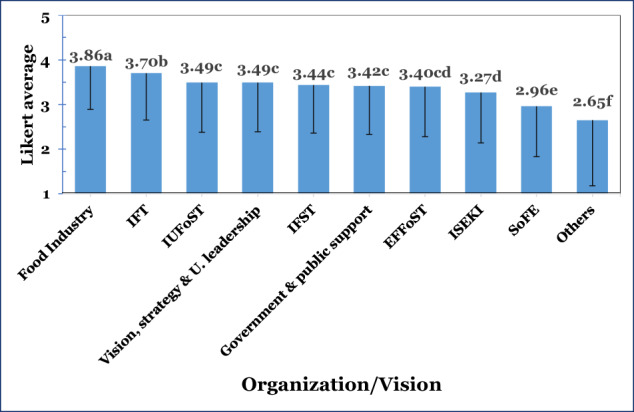
Likert-type averages (1–5 scale) and one side (-) SD of organization/vision impact on FST&E education (values with different letters indicated significant differences between groups; one-way ANOVA with post-hoc LSD test, *p* < 0.05).

The impact of professional organizations on food science/food technology/food engineering education, as well as strategy and vision data, are depicted in Fig. 4.

Data analysis (*t*-test) of the impact of the various organizations or vision and strategy on education revealed that the statistically highest Likert-type average scores (one-way ANOVA with post-hoc LSD test, *p* < 0.05) were given to the ‘Food industry’ (3.86). ‘IFT (Institute of Food Technologists)’ was in the 2nd statistical group (3.70), followed by the 3rd statistical group that included ‘IUFoST (International Union of Food Science & Technology)’ (3.49), ‘Vision, strategy & leadership of the university’ (3.49), ‘IFST (Institute of Food Science+Technology)’ (3.44), and ‘Government, public interest & support’ (3.42). ‘EFFoST (The European Federation of Food Science and Technology)’ (3.40) was between the 3rd and the 4th group that included ‘ISEKI-Food (European Association for Integrating Food Science and Engineering Into the Food Chain),’(3.27). ‘SoFE (Society of Food Engineering)’ (2.96) was the next statistical group, and the last 6th group was ‘Others’ (2.65).

It is quite surprising that the food industry obtained such a high perceived impact on education, especially because the number of respondents in the panel affiliated with academic and educational institutes was high (69.6%). This could be explained by the fact that most curricula are designed to align with the industrial requirement and/or the need to provide students with the essential tools for the food industry. As no in-depth interviews were conducted, these findings warrant additional consideration.

IFT was in second place, significantly affecting FST&E education. In light of the quite low number of respondents from North America and Canada (13.1%), this finding clearly projects the significant role IFT has in impacting global education and proliferation. The 3rd group included IUFoST, IFST (international and mainly UK organizations, respectively), ‘Vision, strategy & leadership of the university’ and ‘Government, public interest & support´. These different groups and elements were perceived as very important and apparently have a significant role in contributing to the education program. EFFoST was categorized between the 3rd and 4th groups, including ISEKI-Food (3.27). These organizations were perceived as lower compared with the previous organizations. SoFE was classified only in the 5th significantly different group. As SoFE appeals mainly to FEs, many panelists were probably unfamiliar with its activities.

### Education impact on professional expectations

**Fig. 5:**
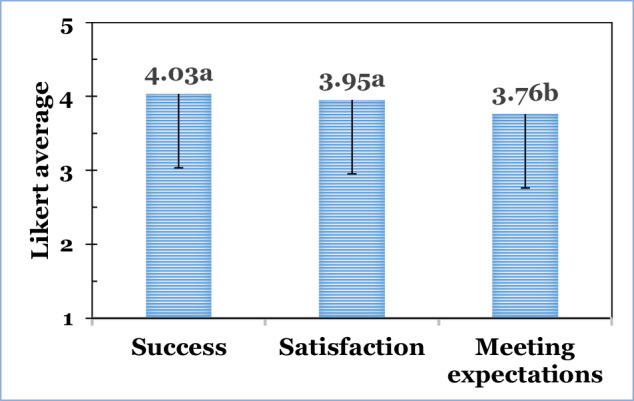
Likert-type averages (1–5 scale) and one side (-) SD of ‘Success’, ‘Satisfaction’, and ‘Meeting expectations’ (values with different letters indicated significant differences between groups; one-way ANOVA with post-hoc LSD test, *p* < 0.05).

The impact of the respondents’ education curricula on their professional success, satisfaction, and meeting expectations data is depicted in Fig. 5.

Education curricula showed two different statistical (one-way ANOVA with post-hoc LSD test, *p* < 0.05) groups. The first group included ‘Success’ (4.03) and ‘Satisfaction’ (3.95). The second statistical group that was quite lower evaluated was ‘Meeting expectations’ (3.76). This finding could open new avenues for education institutes to conduct in-depth assessments of their alumni and graduates, focusing on improving their performances in order to better meet their graduates’ future expectations. This study also provides insights into new education and learning opportunities and new topics to be included in future curricula.

When comparing FSTs with FEs, it was quite surprising that FSTs consistently rated all three attributes lower than FEs. In two cases, these differences were even significant: ‘Success’ (4.07 vs. 4.15, one-way ANOVA with post-hoc LSD test, *p* < 0.05), ‘Satisfaction’ (3.96 vs. 4.06), and ‘Meeting expectation’ (3.78 vs. 3.83, one-way ANOVA with post-hoc LSD test, *p* < 0.05). This lower assessment by FSTs highlights that the potential for curriculum improvements is high, and an in-depth evaluation should open new avenues for significant improvements.

In conclusion, these main points are highlighted:

- Seven topics affecting the future of the profession domain curricula were evaluated between ‘High’ to ‘Very high’. The highest scores were found for: ‘Critical thinking’, followed by ‘Problem-solving projects,’ ‘Teamwork/collaboration’, ‘Innovation/Open innovation’, and ‘Multidisciplinary’.
- The importance of Academic partnership/collaboration showed that ‘Food industry’, and ‘Nutrition’ were ranked the highest.
- Significant positive roles of the food industry in collaboration and partnerships with the FST&E domain were demonstrated. Significant findings were also related to internships, education, strategy, and vision effects of the food industry.
- Collaboration between FST&E and nutrition sciences indicated its high importance. Integrating or converging nutrition science and FST&E is emphasized based on the lack of actual present collaborations.
- Assessing the education curricula contribution showed two statistical groups. The first group included ‘Success’ and ‘Satisfaction’. ‘Meeting expectations’ was the second. New avenues to better meet future graduates’ and students’ expectations were identified.
- Insights into novel education and learning opportunities and new topics to be included in future curricula have been identified.

## Methods

The approach employed encompassed a structured questionnaire, adopting a methodology akin to the one described earlier^12,15^. The questionnaire is provided in the Supplementary information data file. The online questionnaire survey utilized the Qualtrics© software (https://www.qualtrics.com/) and targeted global professionals (including students) across the food sector and nutrition. The key questions were formulated to capture the perspectives on professional values held by individuals in the studied fields. The initial questionnaire was pretested (these data were not utilized in the final analysis) using a pilot sample (*n* = 12) of selected food practitioners from academia and the food industry. This panel was selected based on previous personal and professional interactions. The pilot was employed to ensure the questionnaire’s consistency and to seek suggestions on additional topics that should be incorporated into the revised survey.

The link of the webpage of the questionnaire was distributed by e-mails of numerous organizations (e.g., IUFoST, ISEKI-Food Association, SoFE, IFT) and food practitioners globally. The survey was conducted in English, avoiding any possible language ambiguities. It was completely anonymous and was open from the end of May until the end of July 2022. Both mobile and computerized feedback was offered.

A 5-point Likert-type scale^26^ was applied and consisted of 1 (‘Very low’), 2 (‘Low’), 3 (‘Medium’), 4 (‘High’), and 5 (‘Very high’). For comparisons, the Likert-type scale assessments were transformed into a calculated average. The Likert-type scale is widely employed as a fundamental and commonly utilized psychometric instrument in educational and social sciences research, marketing research, customer satisfaction studies, opinion surveys, and numerous other fields. One topic included ranking (from 1 to 5; each rank could appear only once).

Apart from the professional questions, the survey included demographic information such as gender, age group, location where the most advanced degree was obtained, or current place for study according to the following geographic categories: Western Europe, Eastern Europe, UK, North America including Canada, Mexico, South America, Asia/Middle East, China, Far East (excluding China), Oceania (Australia, New Zealand), and Africa. The questionnaire ended with an open-ended question asking for the interview’s possible suggestions for curriculum improvements. The data were analyzed using Microsoft Excel© spreadsheet (Redmont, Washington), JASP software (ver. 0.16.4, https://jasp-stats.org/), and IBM SPSS Statistics for Windows (version 28; IBM Corp., Armonk, New York). For significant differences (*p* < 0.05) among groups, one-way ANOVA with a post-hoc least significant difference (LSD) test was performed. A two-sided *t*-test was utilized to identify significant differences (*p* < 0.05) between the averages of the two groups.

The survey was written according to the authorization from the Committee for the Use of Human Subjects in Research through The Robert H. Smith Faculty of Agriculture, Food and Environment of The Hebrew University of Jerusalem (file: AGHS/01.15) as outlined previously^12^. Before starting the study, the participants were informed that the responses were completely anonymous. Also, before starting the questionnaire, the consent of the participants was requested, and only those who agreed were able to start the study.

### Reporting summary

Further information on research design is available in the Nature Research Reporting Summary linked to this article.

### Supplementary information

Supplementary information

Reporting summary

## Data Availability

The dataset obtained and analyzed during the current study is available from Prof. Eli Cohen upon request.
