# Author Correction: Emerging challenges and opportunities in innovating food science technology and engineering education

**DOI:** 10.1038/s41538-024-00256-z

**Published:** 2024-02-13

**Authors:** I. S. Saguy, C. L. M. Silva, E. Cohen

**Affiliations:** 1 The Robert H. Smith Faculty of Agriculture, Food & Environment, The Hebrew University of Jerusalem, Rehovot, Israel; 2 Universidade Católica Portuguesa, CBQF—Centro de Biotecnologia e Química Fina—Laboratório Associado, Escola Superior de Biotecnologia, Rua Diogo Botelho 1327, 4169-005 Porto, Portugal; 3 Gilford Glazer Faculty of Business Administration, Ben-Gurion University of the Negev Beer-Sheva, Be’er Sheva, Israel

**Keywords:** Education, Agriculture

Correction to: *npj Science of Food* 10.1038/s41538-023-00243-w, published online 13 January 2024

In the article, incorrect in-text numbered citations were provided in the third paragraph in the section titled “Internships”. The correct text should have appeared as below:

“Bridging the academia-industry gap in the food sector through collaborative courses and internships was recently studied. More than fifteen years of university extension diplomas in food technology Diplomas demonstrated how collaborative courses strengthen academia-industry bonds, and employability was boosted thanks to internships and the network created^22^. Internships could support students in developing their identity, which is achieved by close contact with their future working tasks^34^, enhancing familiarity with and nearness to their future profession^35^ and industry-based projects and governance^36^. Also, student projects in collaboration with the industry make the students face a reality^37^. In light of these benefits, it is clear why the internship in the food industry received such a high Likert-type average. This very high importance given by the panel to industry internships coincides with their ranking, as aforementioned in the previous section, highlighting the core role of the food industry in students’ education.”

