# Supplementary material for: Emerging challenges and opportunities in innovating food science technology and engineering education
Source: NPJ Sci Food. 2024 Jan 13;8:5. doi: 10.1038/s41538-023-00243-w (PMC10786934; doi:10.1038/s41538-023-00243-w)
Supplement: Supplementary file 1 — Supplementary information [file 41538_2023_243_MOESM1_ESM.pdf]

Default Question Block

Dear Colleague/student, this questionnaire is part of a study aiming to:
1. Assess current Food Science & Technology and Food Engineering (FST&E) professional activities and related education programs;
2. Identifying possible challenges and opportunities;
3. Recommending (if needed) some future curricula improvements.
The main outcome will be presented at the 21st IUFoST 2022 Singapore Meeting.
If possible, kindly distribute the link of the questionnaire to your students and/or colleagues.
The responses are completely anonymous and we deeply appreciate your cooperation and precious time. If at any time you have questions, concerns, or suggestions please contact one of us.
THANK YOU,

Prof. Cristina Luisa Silva
Prof. Eli Cohen
Prof. I. Sam Saguy

A. Your consent is required to participate in the survey. Please click the appropriate box to agree to have your data recorded.

- ○ I agree
- ○ I disagree

B. Education - Please select ALL the relevant boxes that relates to your education (tick all boxes that apply):

	Student	BSc/1st Degree	MSc or equivalent	PhD/DSc
1. Food Science/Technology	☐	☐	☐	☐
2. Food Engineering	☐	☐	☐	☐
3. Microbiology	☐	☐	☐	☐
4. Nutrition	☐	☐	☐	☐
5. Chemical Engineering	☐	☐	☐	☐

	Student	BSc/1st Degree	MSc or equivalent	PhD/DSc
6. Bioengineering/Biotechnology	☐	☐	☐	☐
7. Business/Marketing	☐	☐	☐	☐
8. Others (e.g., Agronomy, Agriculture Engineering, Gastronomy Sciences,.......)	☐	☐	☐	☐

C. Profession - What field best describes YOUR main current professional activities (tick only one)

- ○ 1. Food Science and/or Technology (FST)
- ○ 2. Food Engineering
- ○ 3. Microbiology
- ○ 4. Nutrition
- ○ 5. Chemical Engineering
- ○ 6. Bioengineering/Biotechnology
- ○ 7. Business/Marketing
- ○ 8. Consultancy
- ○ 9. Others (e.g., Food Trade Company, Regulators,.....)

D. Affiliation - Which category best describes YOUR CURRENT business affiliation (tick only one):

- ○ 1. Academic/educational institution
- ○ 2. Private research facility
- ○ 3. Food industry
- ○ 4. Foodservice
- ○ 5. Startup/FoodTech
- ○ 6. Government
- ○ 7. Consultancy
- ○ ________ 8. Others, please specify

E. Curricula - How important for YOU are the following topics to be included in developing future curricula

	1. Very low	2. Low	3. Medium	4. High	5. Very high
1. Problem solving projects	○	○	○	○	○
2. Critical thinking development	○	○	○	○	○
3. Entrepreneurship	○	○	○	○	○
4. Creativity	○	○	○	○	○
5. Innovation/open innovation	○	○	○	○	○
6. Teamwork/collaboration	○	○	○	○	○
7. Multidisciplinary	○	○	○	○	○
8. Soft (life) skills	○	○	○	○	○
9. Project/time management	○	○	○	○	○
10. Business creation/network	○	○	○	○	○

F. Academic partnership/collaboration - Please rank the importance for academic programs the following partnership(s) and/or collaboration(s): (from 1=highest to 5=lowest. each rank could appear only once):

	1	2	3	4	5
1. Other academic discipline(s) (e.g., digital/computer science)	○	○	○	○	○
2. Nutrition Sciences	○	○	○	○	○
3. Food Industry	○	○	○	○	○
4. Government, policy makers and/or local authorities	○	○	○	○	○

	1	2	3	4	5
5. Private sector.	○	○	○	○	○

G. Topics - How important are the following topics for FST&E

	1 - Very low	2 - Low	3 - Medium	4 - High	5 - Very high
1. Innovation/open innovation	○	○	○	○	○
2. Startup/FoodTech	○	○	○	○	○
3. Artificial Intelligence, machine learning	○	○	○	○	○
4. Big Data, Communication, Robotics	○	○	○	○	○
5. Entrepreneurship	○	○	○	○	○
6. Sustainability, circular economy, food waste management	○	○	○	○	○
7. Nutrition Sciences	○	○	○	○	○
8. Kaizen methodologies/Continuous improvement	○	○	○	○	○
9. Management/marketing	○	○	○	○	○
10. New product development	○	○	○	○	○
11. Consumer perception & trust	○	○	○	○	○

H. Curricula - How important are the following to FST&E curricula to meet future challenges and learning opportunities

	1 - Very low	2 - Low	3 - Medium	4 - High	5 - Very high
1. Revision of the current education programs	○	○	○	○	○

	1 - Very low	2 - Low	3 - Medium	4 - High	5 - Very high
2. Hybrid (remote and frontal) teaching	◯	◯	◯	◯	◯
3. Apprenticeships (e.g., industrial training)	◯	◯	◯	◯	◯
4. Enhanced integration with nutrition	◯	◯	◯	◯	◯
5. Research project(s)	◯	◯	◯	◯	◯
6. Soft (life) skills	◯	◯	◯	◯	◯
7. Employability	◯	◯	◯	◯	◯
8. Adaptability (e.g., adjusting to change in real time, managing biases, overcome challenges)	◯	◯	◯	◯	◯
9. Business related(e.g., creation, network, partnership, collaboration)	◯	◯	◯	◯	◯

J. Internship – How important for FST&E students are the following internships

	1 - Very low	2 - Low	3 - Medium	4 - High	5 - Very high
1.1. Academic internship	◯	◯	◯	◯	◯
2. Food industry internship	◯	◯	◯	◯	◯

	1 - Very low	2 - Low	3 - Medium	4 - High	5 - Very high
3. Start-up/FoodTech company internship					
4. Other domains/industries					
5. Internship in other countries					

K. Impact - In YOUR opinion, what impact had the organizations below on the food science/food technology/food engineering education

	1 - Very low	2 -Low	3 - Medium	4 - High	5 - Very high
1. IUFoST (International Union of Food Science & Technology)					
2. IFT (Institute of Food Technologists)					
3. IFST (Institute of Food Science+Technology)					
4. EFFoST (The European Federation of Food Science and Technology)					
5. ISEKI-Food (Integrating Safety and Environmental Knowledge Into Food Studies)					
6. SoFE (Society of Food Engineering)					
7. Food Industry					
8. Government, Public interest & support					

	1 - Very low	2 -Low	3 - Medium	4 - High	5 - Very high
9. Compelling vision, strategy & leadership of your university	○	○	○	○	○
10. Others (please specify): [ ]	○	○	○	○	○

L. Success and Satisfaction – In YOUR opinion, what impact your education curricula had on your professional success, satisfaction and meeting your expectations

	1 Very low	2 Low	3 Medium	4 High	5 Very high
1. Success	○	○	○	○	○
2. Satisfaction	○	○	○	○	○
3. Meeting expectations	○	○	○	○	○

M. Gender

- ○ Male
- ○ Female
- ○ Not willing to answer

N. Age group

- ○ 18 to 25
- ○ 26 to 40
- ○ 41 to 55
- ○ Above 55

P. Where have you received your most advanced degree or where you are studying (tick only one)?

- ○ 1. Western Europe

- 2. Eastern Europe
- 3. UK
- 4. North America including Canada
- 5. Mexico
- 6. South America
- 7. Asia/Middle East
- 8. China
- 9. Far East (excluding china)
- 10. Oceania (Australia, New Zealand)
- 11. Africa

T. Suggestions

- Please add any other pertinent information, comments and/or suggestions

Powered by Qualtrics
